# Generation and filtering of gene expression noise by the bacterial cell cycle

**DOI:** 10.1186/s12915-016-0231-z

**Published:** 2016-02-11

**Authors:** Noreen Walker, Philippe Nghe, Sander J. Tans

**Affiliations:** FOM Institute AMOLF, Amsterdam, 1098 XG The Netherlands

**Keywords:** Cell cycle, Gene expression, Growth, Noise, Single-cell microscopy

## Abstract

**Background:**

Gene expression within cells is known to fluctuate stochastically in time. However, the origins of gene expression noise remain incompletely understood. The bacterial cell cycle has been suggested as one source, involving chromosome replication, exponential volume growth, and various other changes in cellular composition. Elucidating how these factors give rise to expression variations is important to models of cellular homeostasis, fidelity of signal transmission, and cell-fate decisions.

**Results:**

Using single-cell time-lapse microscopy, we measured cellular growth as well as fluctuations in the expression rate of a fluorescent protein and its concentration. We found that, within the population, the mean expression rate doubles throughout the cell cycle with a characteristic cell cycle phase dependent shape which is different for slow and fast growth rates. At low growth rate, we find the mean expression rate was initially flat, and then rose approximately linearly by a factor two until the end of the cell cycle. The mean concentration fluctuated at low amplitude with sinusoidal-like dependence on cell cycle phase. Traces of individual cells were consistent with a sudden two-fold increase in expression rate, together with other non-cell cycle noise. A model was used to relate the findings and to explain the cell cycle-induced variations for different chromosomal positions.

**Conclusions:**

We found that the bacterial cell cycle contribution to expression noise consists of two parts: a deterministic oscillation in synchrony with the cell cycle and a stochastic component caused by variable timing of gene replication. Together, they cause half of the expression rate noise. Concentration fluctuations are partially suppressed by a noise cancelling mechanism that involves the exponential growth of cellular volume. A model explains how the functional form of the concentration oscillations depends on chromosome position.

**Electronic supplementary material:**

The online version of this article (doi:10.1186/s12915-016-0231-z) contains supplementary material, which is available to authorized users.

## Background

Single-cell experiments have shown gene expression to fluctuate randomly under constant conditions [1–7], which can have key consequences for the fidelity of signal propagation [8], cell fate decisions [9, 10], and fitness [4, 11–16]. Noise in gene expression is often quantified by the observed cell-to-cell variability in the production rate or concentration of a protein when observing many cells in an isogenic population [1, 17]. Fluctuations in gene expression can be caused by many local and global factors such as random binding events of RNA polymerase [18], fluctuating concentration of ribosomes, or availability of amino acids [4, 19]. The cell cycle has been suggested as a general source of gene expression noise [17, 19], meaning that, in a snapshot of a population, two cells can differ in protein production rate or concentration because they are in different phases of their cell cycle. Alternatively, two cells at the same cell cycle phase can differ because of cell cycle-independent effects. The key aim of this study is to quantify and disentangle these effects in *Escherichia coli*, and to mechanistically understand cell cycle contributions.

Eukaryotes exhibit distinct cell cycle phases that display different levels of growth activity and of DNA replication, which in turn can result in varying expression levels as the cell cycle progresses. Single-cell investigations of *Saccharomyces cerevisiae* have indeed shown quasi-periodic fluctuations of protein expression rates [20] and concentrations [21] in synchrony with the cell cycle. The prokaryotic cell cycle does not display such distinct replication and growth phases. *E. coli*, for instance, grows and replicates DNA continuously throughout its cell cycle, though for slow growth there are periods without replication activity [22, 23]. Expression activity can be dependent on the cell cycle nonetheless, for example because the replication of a gene may double the transcriptional activity at a specific moment in time, as suggested by recent single-cell studies [17, 24, 25]. That doubling would then in turn affect enzyme concentration and could cause quasi-periodic fluctuations. However, at the same time, cells may exploit specific regulatory mechanisms to filter such perturbations [26, 27]. Direct experimental investigations of the impact of the bacterial cell cycle on expression variability are lacking. Elucidating this question is important to understand the origins of gene expression noise, modeling of genetic circuits, and resulting impact on growth variability [28] as well as other forms of cellular heterogeneity [10].

To address these questions, we followed a single-cell approach. We imaged *E. coli* cells as they grew into micro-colonies and measured gene expression as the fluorescence signal of chromosomally encoded fluorescent proteins (Additional file 1: movie S1). As shown herein, understanding the temporal dynamics requires detailed information on cellular volume increases in time, as protein concentrations are affected both by time-dependent expression and dilution. Thus, we accurately determined protein expression and cell size at sub-cell cycle resolution. We further developed a model to predict the cell cycle dependence and amplitude of these quasi-periodic fluctuations in expression rate and concentration. The model predicted their dependence on chromosomal position, which we tested with genetic constructs.

## Results and discussion

### The protein production rate fluctuates quasi-periodically

**Fig. 1:**
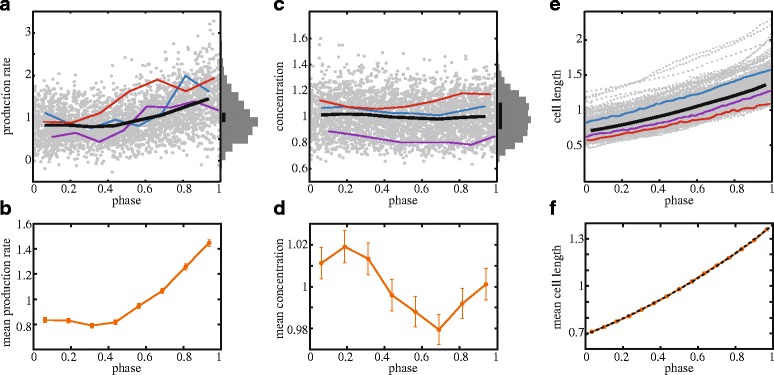
Dependence of protein production rate (**a**, **b**), protein concentration (**c**, **d**) and cell length (**e**, **f**) on cell cycle phase. Observables are normalized by the respective population average and therefore unitless. (**a**, **c**, **e**) Data for 393 cells (*gray*) with three example traces and the binned colony average (*thick black line*). Histograms display the total frequency of production rate or concentration values summed over all phases. To convey the differences in noise intensity, a bar of size 0.2 times the population mean is displayed. (**b**, **d**, **f**) Phase-dependence of the binned data. In (**f**) an exponential function (*black dashed line*) is fitted to the averaged cell length. Error bars are obtained by bootstrapping. For cell length, error bars are plotted but are smaller than the line thickness. Growth was on minimal medium supporting a growth rate of 0.6 dbl/h

To measure the effect of the cell cycle on protein expression, we first determined protein production rate as quantified by the time derivative of the total cellular fluorescence (Methods). Taking the data for all cells with a completed cell cycle (n = 393) over all cell cycle phases, the protein expression rate displayed a total noise intensity (defined as standard deviation divided by the mean) of 0.48 [17]. When plotting the production rate versus cell cycle phase *ϕ* (where 0 is cell birth and 1 is cell division) and averaging over all cells (Fig. 1a), it displayed the following trend: it was approximately constant in the first half, after which it rose to about two-fold at the end of the cycle (Fig. 1b, Additional file 2: Figure S1). An initially constant rate and two-fold increase is consistent with the known chromosome replication pattern for the observed mean growth rate (0.6 dbl/h): a single chromosome copy in the first period of the cell cycle, after which replication occurs in the second period that produces two copies [29]. Each chromosome copy then yields a fixed expression rate. This is not unreasonable, as other components required for expression, such as RNA polymerases and ribosomes, also double throughout the cell cycle. At faster growth, replication occurs throughout the cell cycle for multiple nested chromosome copies [30]. Consistently, we found that the production rate was not initially flat, but instead rose continuously throughout the cell cycle when growing on a different medium that supported a higher mean growth rate of 1.8 dbl/h (Additional file 2: Figure S2). The total increase remained two-fold, in agreement with an expected doubling of the number of gene copies. Overall, these data indicate that the mean protein expression rate is likely proportional to the gene copy number and hence doubles during chromosome replication. This variation is more continuous at high growth rate because of the nested replication and overall higher gene copy numbers.

### Deterministic cell cycle variations contribute to expression noise

To quantify the contribution of the mean cell cycle fluctuations (Fig. 1b) to protein production noise we split the single-cell production rate (which is distinct from the protein concentration) *p*(*ϕ*, **x**) into the population averaged rate $\overline{p_c}\left(\phi \right)$ and individual deviations *δp*(*ϕ*, **x**), which together capture all cell-to-cell variability (Fig. 1a,b): $p\left(\phi, \mathbf{x}\right)=\overline{p_c}\left(\phi \right)+\delta p\left(\phi, \mathbf{x}\right)$. Here, *ϕ* denotes the cell cycle phase and **x** all other causes of cell-to-cell variability; *c* refers to cell cycle dependence, which here is redundant because it is implied by the *ϕ* dependence but used for notation consistency. $\overline{p_c}\left(\phi \right)$ can be estimated by the curve in Fig. 1b, and subtracted from individual traces to obtain an estimate for *δp*(*ϕ*, **x**). The noise intensity caused by the deterministic cell cycle fluctuation $\overline{p_c}\left(\phi \right)$ is 0.26, which was obtained by considering the phase *ϕ* as a random variable and then calculating the variance of the trace. Noise of the individual expression traces *δp*(*ϕ*, **x**), averaged over all cells and *ϕ*, was 0.42 (Additional file 2: Figure S3a). These values are consistent with a scenario in which population mean trace $\overline{p_c}\left(\phi \right)$ and deviation traces *δp*(*ϕ*, **x**) are independent and thus their variances (squared noise) can be added up: 0.48^2^ ≈ 0.26^2^ + 0.42^2^. This population-average cell cycle contribution towards production rate noise does not include cell cycle stochasticity of individual cells and we will consider that below.

### Concentration fluctuations are buffered by dilution

Fluctuating production rates can cause noise in the protein concentration. To determine the latter, we quantified the mean fluorescence per unit area of the cell. The noise intensity of 0.15 (0.10 for fast growth), which was obtained by taking the data of all cells and at all cell cycle phases, was consistent with previous reports [1]. After ordering by cell cycle phase and averaging (Fig. 1c), the concentration also showed systematic variations (Fig. 1d*,* Additional file 2: Figure S1): it increased slightly right after cell birth, then decreased and finally rose again. The amplitude of these variations was 4 % of the mean. This low value (Additional file 2: Figure S3b) and the initial increase seemed inconsistent with the large amplitude of variations in production rate caused by the cell cycle, as well as with the initially constant value of production rate (Fig. 1b) [25].

**Fig. 2:**
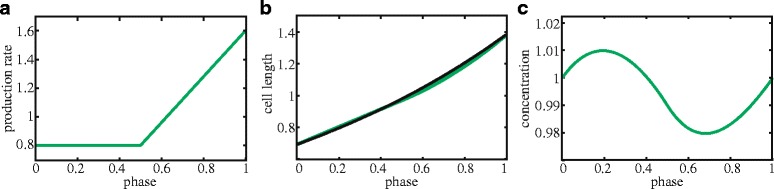
Model for cell cycle dependence of protein concentration. **a** Average protein production rate normalized by the mean. **b** Exponential length increase normalized to a mean of one (*black*). Population average protein production rate integrated in time, or the population average total fluorescence (*green*). **c** Determined cellular protein concentration, given by the green signal divided by the *black line* in panel b

To get a more intuitive understanding of these differences, we formulated a minimal cell cycle model based on the measured cell cycle dependency of production rate (Fig. 1b). The concentration cannot be determined by simply integrating the production rate, as this would ignore dilution due to volume growth. To quantify the volume growth, we determined for each cell its length and its dependence on the cell cycle phase (Fig. 1e, Methods) [28]. The population mean cell length $\overline{L}\left(\phi \right)$ was well described by an exponential function (Fig. 1f) [31–33], and not by bi-linear or linear functions (Additional file 2: Figure S4), as suggested previously [34–37]. Therefore, an exponential function for cell size was used as input for the minimal model (Fig. 2b). With a mean protein production $\overline{p}\left(\phi \right)$ at phase *ϕ* (Fig. 2a), the concentration $\overline{E}\left(\phi \right)$ can then be written as: $\overline{E}\left(\phi \right)=\left(\overline{F_0}+{\displaystyle {\int}_0^{\phi }}\overline{p}\left({\phi}^{\hbox{'}}\right)d{\phi}^{\hbox{'}}\right)/\overline{L}\left(\phi \right)$, where $\overline{F_0}$ is the total amount of protein at cell birth.

By design, $\overline{E}\left(\phi \right)$ (Fig. 2c) reproduced the measured data (Fig. 1d) and provided an explanation for the observed functional form, including the counterintuitive increase in concentration at the beginning of the cell cycle, before duplication occurs. As $\overline{E}\left(\phi \right)$ is periodic, we know that increases (dilution rate smaller than expression rate) are balanced by decreases (dilution rate larger than expression rate). In cases where duplication occurs late, the expression rate is essentially constant because there is only one gene copy, while the dilution rate changes. Thus, dilution is then comparatively weak at the beginning of the cell cycle, resulting in an increasing concentration, while dilution is comparatively strong further into the cell cycle, resulting in decreasing concentrations. This rationale also explains why concentration fluctuations are small: the functional form of the total fluorescence (as a function of the cell cycle phase) is almost identical to that of the volume (Fig. 2b).

### Stochastic replication timing contributes to expression noise

**Fig. 3:**
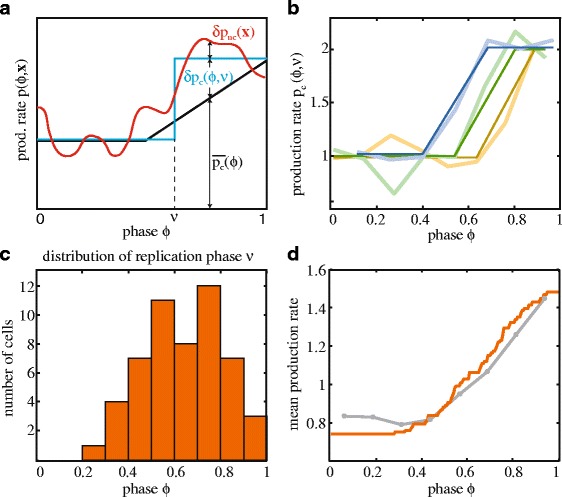
Production rates of single cells. **a** Description of variables used for noise decomposition. The protein production rate *p*(*ϕ*, **x**) (*red line*) is the sum of three contributions: (1) the population-average cell cycle fluctuations $\overline{p_c}\left(\phi \right)$ (*black line*), (2) the contribution due to stochastic replication timing (difference between *blue* and *black line*, *δp*_*c*_(*ϕ*, *ν*)), and (3) stochasticity resulting from other, unknown, noise sources (difference between *red* and *blue line*, *δp*_*nc*_(**x**)). The sum of *δp*_*c*_(*ϕ*, *ν*) and *δp*_*nc*_(**x**) represents all of the stochastic contributions *δp*(*ϕ*, **x**). The phase at which replication occurs is denoted by *v*. **b** Experimental traces of three different cells (*thick lines*) and fitted step functions (*thin lines*). See Additional file 2 for definition of step function. Initial value was set to 1 and data is slightly vertically shifted for clarity. **c** Histogram of *v*. Data is from 53 cells in which a step-function could be discerned from the rest of the noise (13.5 % of the traces). **d** Comparison of experimental average production rate curve (*gray line*) and mean of ideal step functions (*orange line*). Experimental curve is the same as in Fig. 1b, which was obtained from binning the data according to cell cycle phase and averaging over n = 393 cells

The single cell data also suggested that stochasticity in replication timing is a source of protein production noise, which is supported by previous studies [23, 38] (Fig. 1a, thin lines). In other words, *δp*(*ϕ*, **x**) would be the sum of fluctuations caused by cell cycle stochasticity *δp*_*c*_(*ϕ*, *ν*) and of fluctuations *δp*_*nc*_(**x**) unrelated to the cell cycle (Fig. 3a). Here, *v* is the cell cycle phase at which the gene of interest is replicated and *v* varies from cell to cell. Thus, the sum of *δp*_*c*_(*ϕ*, *ν*) and the population-average $\overline{p_c}\left(\phi \right)$ yield all the fluctuations *p*_*c*_(*ϕ*, *ν*) caused by the cell cycle. To determine the stochastic contribution of the cell cycle to the expression noise, one needs to quantify *δp*_*c*_(*ϕ*, *ν*). However, it is not trivial to distinguish *δp*_*c*_(*ϕ*, *ν*) from the other stochastic, non-cell cycle variations in the experimental single-cell traces.

To overcome this problem, we started with *p*_*c*_(*ϕ*, *ν*) and followed a variance decomposition approach using the law of total variance [39, 40]. The variance of the full cell cycle fluctuations can be decomposed as follows:

1 $$ Var\left({p}_c\left(\phi, \nu \right)\right)=\left\langle Var\left({p}_c\left(\phi, \nu \right)\Big|\phi \right)\right\rangle +Var\left(\left\langle {p}_c\left(\phi, \nu \right)\Big|\phi \right\rangle \right) $$

Here, angular brackets denote averaging, and the notations *Var*(… |*ϕ*) and 〈 … |*ϕ*〉 indicate, respectively, the variance and the average for a given phase *ϕ* (conditioned on *ϕ*). In the second term, the brackets thus indicate an averaging over the stochastic variable *v*, which yields $\overline{p_c}\left(\phi \right)$. Next, the variance is taken. This variance was in fact calculated previously, and found to be (0.26)^2^ (Fig. 1b). Thus, the second term indicates the deterministic contribution to the cell cycle induced noise.

In the first term, the variance of *p*_*c*_(*ϕ*, *ν*) is determined conditionally on *ϕ*, and then averaged. This term thus denotes the stochastic contribution to the cell cycle-induced noise. The data does not directly provide an estimate of this variance, because the cell cycle-induced noise and noise from other sources are confounded in the measured single-cell traces of the production rate (Fig. 1a). Indeed, in these traces, other noise sources, such as metabolism [28] and fluctuating transcription factors [1], are substantial and can mask the quick two-fold increase expected from gene replication events. However, in a subset of traces, the two-fold increase was clear (Fig. 3b,c, Methods). Fitting each of these traces with a step-function (Additional file 2: Figure S5) provided a distribution of the step-moment, *v*. We obtained a wide distribution for *v* with a mean 0.64 and a standard deviation of 0.17 (Fig. 3c). To check whether this distribution was consistent with the full dataset, we compared the average of the fitted step-functions to the average of all measured traces ($\overline{p_c}\left(\phi \right)$, Fig. 1b), and found that they were similar (Fig. 3d). These findings suggested that gene duplication events with stochastic timing in individual cells underlie the smooth shape of the population average production rate (Fig. 1b).

The distribution of *v* (Fig. 3c) now allowed us to estimate the first term in eq. (1), by first determining the variance of the step-functions at fixed phase, and then averaging over all phases (Additional file 2: Figure S6a). We obtained a value of (0.23)^2^ for this stochastic contribution of the cell cycle to expression noise, which is comparable in magnitude to the deterministic contribution denoted by the second term ((0.26)^2^, Additional file 2: Table S1). Thus, variability in initiation timing contributes substantially to the cell cycle-induced noise. The deterministic and stochastic contributions together (*p*_*c*_(*ϕ*, *ν*)) thus caused a variance of (0.23)^2^ + (0.26)^2^ = (0.35)^2^, which is about half (52 %) of the protein production variance (Fig. 5b, Additional file 2: Table S1).

To estimate how the protein concentration noise is affected by the cell cycle, we computed the concentration traces resulting from the step-like production rate functions (Additional file 2: Figure S6a). For each *p*_*c*_(*ϕ*, *ν*) of the set (Fig. 3c) the corresponding concentration curve was computed, considering that proteins are diluted due to volume growth (Additional file 2: Figure S6b). We found that the quasi-periodic concentration fluctuations caused by the cell cycle (which includes deterministic and stochastic components) contributed less than 1.5 % to the variance in protein concentration (Additional file 2: Figure S6b and Fig. 5b). Note that one can distinguish contributions from the population average trend (Fig. 1d) and the stochastic deviations around it due to variability in replication timing (less than 1 % contribution each, Additional file 2: Table S1).

### Location on the chromosome affects expression fluctuations

**Fig. 4:**
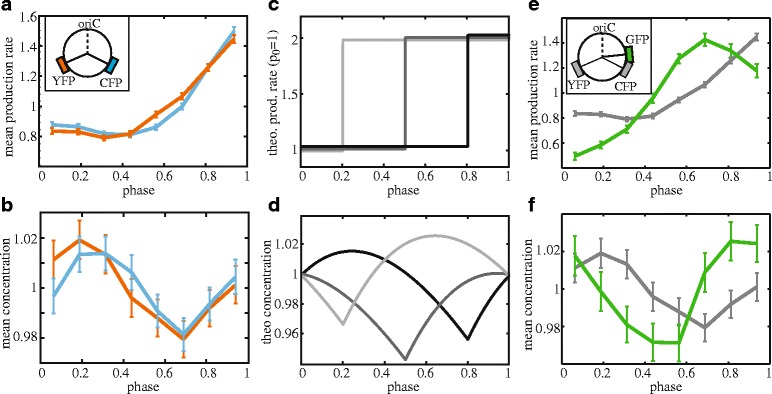
Influence of chromosomal position. Production rate (**a**) and concentration (**b**) for genes at equidistant and symmetric positions with respect to the origin of replication. Observables were normalized by the population mean. (Inset) Locations of fluorescent genes and origin of replication *oriC* on the DNA. Different replication times (**c**, *light* to *dark gray*) and their predicted influence on the concentration (**d**). Production rates are slightly vertically shifted for clarity. (Normalized) production rate (**e**) and concentration (**f**) of GFP (strain ASC636, *green line*) compared to YFP (strain MG22, *gray line*). GFP data is from 296 cells with complete cell cycle that have on average seven data points/cell cycle. (Inset) Location of *gfp* compared to other fluorescent genes. Error bars are obtained by bootstrapping

Chromosome replication is initiated at the origin of replication (*oriC*) from which two replication forks then progress simultaneously and bi-directionally along the two strands of DNA [41]. This raises two expectations: first, genes located at opposite sides but at the same distance from *oriC* should be duplicated at the same time and thus show the same cell cycle dependence of protein production and concentration. Second, if one gene is located upstream of the other, the increase in protein production should occur earlier. To test the first prediction, we investigated a *cfp* gene positioned symmetrical to the *yfp* gene studied so far, at the opposite strand at the same distance from *oriC* (Methods, Fig. 4a inset). We indeed found that both reporters displayed a similar dependence of production rate and concentration on cell cycle phase (Fig. 4ab, Additional file 2: Figure S1).

To change the position we studied a *gfp* gene under *P*_*lac*_ control closer to *oriC* than *yfp* or *cfp* (Methods, Fig. 4e). As expected from the earlier replication, the GFP production rate indeed increased earlier than the previous YFP signal (Fig. 4e). It started comparatively low, then increased more than two-fold and subsequently decreased again to end at twice the initial rate (Fig. 4e). The cause of the high fold-change and decrease is unknown, but changes in chromosome structure or transient improvement in competition for RNA polymerases for this promoter (two binding sites at the two replicated genes) could play a role. As predicted by the model (Fig. 4c,d), the dip in GFP concentration occurred earlier and the initial increase disappeared (Fig. 4f). The magnitude of fluctuations remained at around 4 %. Overall, these data show that gene position on the chromosome affects cell cycle-related noise.

## Conclusions

**Fig. 5:**
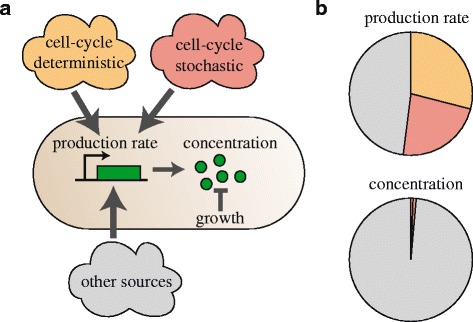
Summary of observed contributions to gene expression noise. **a** The cell cycle causes fluctuations in the protein production rate, through deterministic and stochastic contributions. Other non-cell cycle-related sources contribute as well. The fluctuations in protein concentration are determined by protein production noise and dilution due to growth. **b** Contributions of the different noise sources, as described in panel a, as fractions of the total observed variance in gene expression (Additional file 2: Table S1)

In summary, we found that the cell cycle can be a major causal factor of observed noise in the rate of gene expression (52 %), with the rest coming from other sources such as metabolism [28, 42, 43], transcription factors [8], or expression machinery [18] (Fig. 5a). Within the cell cycle contribution, the data suggests two components: a deterministic mean determined by the cell phase (29 %), and a stochastic contribution caused by variability in the timing of replication (23 %) (Fig. 5b). The initially flat production rate suggested gene copy number is the main factor in cell cycle-induced expression rate variations, though alternative factors, such as cell cycle-induced variations in transcription factor concentrations, could also contribute.

The analysis indicated a noise-cancelling mechanism: even sudden two-fold production rate increases caused by replication of the gene are effectively compensated for by an acceleration of dilution due to exponential growth [26, 27] (Fig. 5b). The observed minor effect of the cell cycle on the protein concentration is thus due to a passive homeostasis mechanism that exploits the balance between synthesis and dilution. When proteins are actively degraded, this noise cancelling mechanism would be less efficient. We note that a similar, but likely active, balancing between synthesis and dilution was observed in mammalian cells where transcription rate is adjusted to cell size [44, 45]. The homeostatic mechanism we observed does not necessarily act on noise from other sources, such as fluctuations in RNA polymerase availability [18] or transcription factors [2], if they are not synchronized with exponential volume growth. Indeed, concentrations do display significant noise intensities (0.15 for slow growth, 0.10 for fast growth). We note that other cancelling mechanisms can act on non-cell cycle expression noise. For instance, metabolic noise that causes expression noise is partially compensated for by increased growth [28].

Our findings provide insight into how elementary processes, such as gene replication events and volume growth, can cause and filter noise in bacterial cells. Elucidating the sources of gene expression noise is important to obtaining a bottom-up understanding of cellular heterogeneity, cellular homeostasis, and cell cycle regulation, and to providing input for mathematical models of gene expression networks. Our results confirm previous demonstrations that variance decomposition can be a useful tool in disentangling different noise sources within cells.

## Methods

### Strains, growth media, and cell growth

*E. coli* strain MG22 [1] was used for all experiments unless noted otherwise. This strain is a derivative of MG1655 that contains *yfp* and *cfp* under control of a *lac* promoter, which were inserted into the chromosome at the *intC* and *galK* locus. These two loci are equidistant from the origin of replication, on opposite halves of the circular chromosome. Additionally, we used strain ASC636 in which gene *lacA* of the *lac* operon was replaced by *gfp* (constructed by A. Böhm).

For microscopy experiments we used either M9 minimal medium (main text figures) or rich defined medium (MOPS EZ rich defined medium from Teknova, Additional file 2: Figure S2). M9 was supplemented with 200 μM uracil and 0.1 % maltose was added as a carbon source, yielding a growth rate of 0.6 dbl/h. To the rich medium we added 0.2 % glycerol as a carbon source, yielding a growth rate of 1.8 dbl/h. In all experiments we also added 200 μM IPTG to fully induce the *lac* promoters.

Cells were inoculated in the morning from −80 °C glycerol stock into tryptone yeast (TY) medium and grown for 7 h at 37 °C. Then, cells were diluted highly (~1/10,000 to ~1/100,000) to three different final concentrations into the defined experimental medium (see above). TY was thereby diluted to <0.05 vol% and remaining TY was consumed by the cells. Cells were grown overnight and the next morning a falcon tube with still exponentially growing cells (OD600 < 0.2) was chosen. Cells were diluted again to OD600 = 0.005 and then used for microscopy.

### Sample preparation and microscopy

The sample preparation for microscopy was done in the warm room at 37 °C to minimize temperature stress. We placed a polyacrylamide gel into a glass chamber, pipetted 1 μL of cells onto the gel and closed it with a coverslip. The sample was sealed with a metal clamp (Additional file 2: Figure S7a). This setup provides an oxygen reservoir for the cells but avoids gel dehydration.

To produce polyacrylamide gels, we mixed 1.25 mL 40 % acrylamide, 3.7 mL water, 50 μL fresh 10 % ammonium persulfate, and 5 μL TEMED, poured it into a glass chamber and let it polymerize [28, 46]. Then, the gel was cut into pieces of ~1 cm^2^ and stored in a falcon tube with water (Milli-Q). Before the experiment a gel pad was placed into a falcon tube with 5 mL of the respective growth medium and the medium was exchanged several times.

Cells were imaged with a Nikon TE2000-E inverted microscope using a 100× oil objective (Nikon, Plan Fluor NA 1.3) and additionally 1.5× intermediate magnification. It was equipped with a cooled CCD camera (Photometrix, Cool-Snap HQ), a Xenon arc lamp with liquid light guide (Sutter, Lambda LS) for fluorescence illumination, computer controlled shutters (Sutter, Lambda 10–3 with SmartShutter), and an automated stage (Märzhäuser). The microscope was located in an incubation chamber (Solent) to keep the temperature at 37 °C. Fluorescence filters were obtained from Chroma and we used #49003 (YFP), #49001 (CFP), and #41017 (GFP). Microscope and image acquisition were computer controlled with MetaMorph software (Molecular Devices).

We searched the pad for isolated cells and manually saved the positions of 4–6 cells. Then, an automated script was looped repeatedly (for up to 20 h) over all positions and images of the growing microcolonies were acquired. Loop times were adjusted to cell doubling times and we used 100 sec/loop for μ = 0.6 dbl/h (main text figures) and 45 sec/loop for μ = 1.8 dbl/h (Additional file 2: Figure S2). Each loop, the routine refocused based on image contrast (Brenner algorithm) and acquired phase contrast images at three different heights (±0.2 μm off focus and in focus). Fluorescence images were acquired approximately every 7 loops to reduce photodamage and bleaching. This resulted in 55 phase contrast and 8 fluorescence images per cell cycle, on average. Fluorescence images were 2 × 2 binned to increase signal-to-noise ratio and illumination was kept as short as possible (YFP: 25 ms, CFP: 30 ms, GFP: 50 ms). Growth rates under the microscope were very similar to bulk growth rates in a plate reader.

Movies were obtained until cells formed a second layer or outgrew the field of view, which happened usually after 8–9 generations and a colony size of several hundred cells. Each experiment was performed at least twice (Additional file 2: Figure S1). Data of YFP and CFP (which are encoded in the same strain) that is displayed in a single figure was obtained from the same microcolony in the same experiment (e.g. Fig. 4ab, Additional file 2: Figure S1 and Figure S2). The replicate displayed in Additional file 2: Figure S1 is a different experiment than Fig. 1. Experiments at different growth rates (e.g. Fig. 4ab versus Additional file 2: Figure S2) are also independent experiments. GFP is encoded in a different strain, and hence the GFP data was measured in different experiments than the YFP and CFP data.

### Image analysis

Images were analyzed with custom MATLAB software (MathWorks) based on Schnitzcells [47]. An automatic script determined cell outlines by applying a Laplacian of Gaussian filter to the averaged phase contrast image and then cut accidently connected groups of cells based on the concavity of the cell outline (Additional file 2: Figure S7b). Segmentation was checked manually and corrected if necessary. Cells were then tracked from image to image, resulting in a branch-structured lineage tree, and tracking was checked manually. For all cells with an observed complete cell cycle each measured time point was associated with a cell cycle phase, with 0 being cell birth and 1 cell division.

Cellular length was used as measure for cell size because the rod shaped *E. coli* only grow along their long axis and cell width is independent of cell cycle phase (Additional file 2: Figure S8, ref. [48], contrary to results in ref. [49]). We fitted a third degree polynomial through the silhouette of the segmented cell [28]. The cell length was then obtained by integrating the curve between both cell poles.

Fluorescence images were corrected for alignment offset, background (camera noise), uneven illumination, and were deconvolved to suppress blur (Additional file 2: Figure S7c). To obtain autofluorescence intensity we measured a non-fluorescent strain (MG1655) with the same illumination settings as standard experiments. Measured signal intensity was 2.5 % (CFP) resp. 0.4 % (YFP) of the actual concentration signal from fluorescent proteins. Fluorescence signals were not corrected for autofluorescence because this was small compared to the signal and only introduced a constant offset with no effect on the results.

The total fluorescence of each cell was determined by summing up the pixel intensities within the cellular outline. The protein production rate p(t) was determined as the slope of a linear fit of three consecutive total fluorescence data points centered around t. For the first and last measurement of the cell cycle, fluorescence information of parent and daughter cells, respectively, was used to determine the slope. Protein concentrations were calculated by dividing total fluorescence by the segmented cellular area.

### Data processing

We analyzed cell cycles within a time window of the experiment that showed a constant population-mean growth rate and mean protein concentration. Population mean growth and concentration were considered constant when they fluctuated less than 5 % around the long-term average (for GFP in strain ASC636 10 % was used as cutoff criterion). Cells that stopped growing or were filamented were removed (less than 15 cells per dataset). The fraction of analyzed cells relative to all cells observed with a complete cell cycle was over 86 % for MG22 datasets and over 50 % for ASC636 datasets. The main conclusions were robust to taking the complete data set of growing non-filamentous cells with complete cell cycles. Datasets contained between 215 selected cells (large cells in rich medium) to 435 selected cells (minimal medium). If one dataset was used for multiple plots (e.g. Figs. 1 and 4a,b), the same cells were analyzed.

Traces of production rate (Fig. 3) for individual cells were considered ‘step-traces’ when they deviated from a fitted step-trace (see also Additional file 2) less than a fixed threshold. To be considered a step-trace, the mean squared deviation of a data point on the trace from the fitted value had to be below 2 % of the squared trace-average. Figures and percentages in the main text are determined from one microcolony per strain and growth condition. Results for the repeat experiment are shown in Additional file 2: Figure S1.

To determine the average dependence of a signal (e.g. concentration) on cell cycle phase we binned the signal according to phase and averaged it within each bin (Fig. 1b,d,f). Error bars are standard errors of the mean from a resampled distribution of the signal, obtained by bootstrapping from the experimental data for each bin.

The contribution of a specific noise source *X* (for example, deterministic cell cycle variations) to total protein production noise was calculated by using the additivity of variances for independent variables. The production rate *p* was, for example, written as sum *p* = *X* + *Y* with *Y* being the unknown, unmeasured, fluctuations (such as *δp*(*ϕ*, **x**), see also main text). Then, *Var*(*p*) = *Var*(*X*) + *Var*(*Y*), and the fraction of the variance in *p*, which is caused by *X*, is *Var*(*X*)/*Var*(*p*). We normalized variables such as *p* by their mean so that the squared noise is identical to the variance. For example, the contribution of $\overline{p_c}\left(\phi \right)$ to *p*(*ϕ*, **x**) is 0.26^2^/0.48^2^. For protein concentration, the calculation is identical.

## Additional files

Additional file 1:
**Movie S1.** Time lapse movie of growing cells. Phase contrast and *yfp* fluorescence channel for MG22 cells grown on M9 + 0.1 % maltose + 200 μM Iptg (for example Fig. 1). The movie covers a time of 12 h 40 min. (AVI 863 kb) Additional file 2: Figures S1-S8, Table S1. Supplementary methods. (DOCX 1071 kb)
