# Supplementary material for: Generation and filtering of gene expression noise by the bacterial cell cycle
Source: BMC Biol. 2016 Feb 11;14:11. doi: 10.1186/s12915-016-0231-z (PMC4750204; doi:10.1186/s12915-016-0231-z)
Supplement: Additional file 2: Figures S1-S8, Table S1. — Supplementary methods. (DOCX 1071 kb) [file 12915_2016_231_MOESM2_ESM.docx]

**SUPPORTING ONLINE MATERIAL**

Generation and filtering of gene expression noise by the bacterial cell cycle.

Noreen Walker, Philippe Nghe and Sander J. Tans

**Fitting bacterial growth with an exponential, linear and bilinear function**

Since the precise law of bacterial growth is important for cell-cycle fluctuations of protein concentration, we assessed fits of different growth functions to the binned cellular length data. We examined exponential [1–3], linear [4, 5] and bilinear [6, 7] fit functions for growth. Goodness of fit was evaluated via the mean square error $MSE$ which is the average squared deviation of a data point from the fitted curve:

.

Here, is the distance of a data point from the fitted value, $n$ is the number of data points and the sum runs over all data points. Average length of each cell is normalized to one. More elaborate measures which introduce a penalty for increasing number of fitting parameters (four vs. two free parameters for bilinear vs. exponential fit) gave essentially the same result.

The best fit to the population average function of cellular growth was an exponential function (Fig. S4, $MSE=7.4\cdot10^{-6}$). A bilinear fit was slightly worse ($MSE=31.1\cdot10^{-6}$), while cellular growth clearly does not follow a linear growth law ($MSE=316.8\cdot10^{-6}$). For several single-cell individual traces, a bilinear fit was better than an exponential fit. Since our analysis holds for exponential and bilinear growth functions and an exponential growth law may be more physiologically relevant we chose an exponential function.

**Fit of step functions to production rate traces**

We determined production rate as the slope of a linear fit to three subsequent total-fluorescence data points. This discrete derivative smoothens a theoretical perfect step-wise increase of production rate and makes the increase appear more gradual. In order to fit step functions to production rate traces (Fig. 3 in the main text), we needed to know what a perfect and immediate step function would look like after our data processing. We used this result to fit the single cell traces in the main text.

Let $d_{1}$, $d_{2}$, …,$d_{n}$ be the phase points for which fluorescence data exists ($n$ fluorescent data points for one cell cycle, $0\leq d_{i}\leq1$) (Fig. S5, a and b). All data points have an equal phase difference of $\Delta d=d_{(i+1)}-d_{i}$. The sudden increase of production rate is supposed to occur at phase $x$ which is in between $d_{i}$ and $d_{(i+1)}$ but may also be on either boundary of this interval. $\Delta x$ is the phase between the step time and the *next* data point $d_{(i+1)}$. The initial production rate is $p_{0}$ and doubles at $x$ to be $2p_{0}$.

For calculation of production rate at phase point $d_{k}$ the total fluorescence data $F_{(k-1)}$, $F_{k}$ and $F_{(k+1)}$ at phase $d_{(k-1)}$, $d_{k}$ and $d_{(k+1)}$ has to be fitted by a linear function under a least square criterion. For $k<i$, resp. $k>(i+1)$ the fitted production rate is simply $p_{0}$, resp. $2p_{0}$. However, for the two data points close to the transition in production rate, i.e. at phase $d_{i}$ and $d_{(i+1)}$, a smoothing effect will occur. For calculation of production rate at these points we need the total fluorescence at the following phase points:

$$\begin{matrix} \text{phase total fluorescence} \\ d_{(i-1)}\text{ }F_{(i-1)} \\ d_{i}\text{ }F_{i}=F_{(i-1)}+p_{0}\Delta d \\ d_{(i+1)}\text{ }F_{(i+1)}=F_{(i-1)}+p_{0}\Delta d+p_{0}\Delta d+p_{0}\Delta x \\ d_{(i+2)}\text{ }F_{(i+2)}=F_{(i-1)}+2p_{0}\Delta d+p_{0}\Delta x+2p_{0}\Delta d \end{matrix}$$

Fitting a linear function to $F_{(i-1)}$, $F_{i}$, $F_{(i+1)}$ to obtain the production rate $p(d_{i})$, resp. to $F_{i}$, $F_{(i+1)}$, $F_{(i+2)}$ to obtain $p(d_{(i+1)})$, we get (see Fig. S5 c):

$$\begin{matrix} p(d_{i})=p_{0}\cdot(1+0.5\Delta x/\Delta d) \\ p(d_{(i+1)})=p_{0}\cdot(1.5+0.5\Delta x/\Delta d) \end{matrix}$$

Thus, after data processing, we obtain one (Fig. S5 c, blue and green line) or two (red line) data points with intermediate production rate. Our time resolution is set by the acquisition frequency of fluorescent images and we therefore restricted the potential phases for step-events to these time points (i.e. $\Delta x=0$). Therefore, we fitted single cell traces with a smoothed step function that contains one intermediate data point at $1.5p_{0}$ (Fig. S5 c, blue and green line).

We found that the fitting procedure did not allow reliable detection of doubling events within the first 25 minutes after cell birth, e.g. because of the resulting limited number of data points before the doubling. Including or excluding such potential early doubling events gave similar results however (Fig. S9). In the main text analysis the potential early events are excluded. Note that given the position of the gene on the chromosome and the slow growth rate [8], such early events are not expected.

**Supplementary Table**

|  | a)  Total variance | b)  Variance of deterministic cell-cycle fluctuations | c)  Variance caused by stochastic replication timing | Total variance due to cell-cycle fluctuations:  b)+c) | Variance caused by non-cell-cycle effects:  a)-b)-c) |
| --- | --- | --- | --- | --- | --- |
| Production rate | 0.48^2^ | 0.26^2^ | 0.23^2^ | 0.35^2^ | 0.33^2^ |
| Concentration | 0.145^2^ | 0.011^2^ | 0.013^2^ | 0.017^2^ | 0.144^2^ |

**Table S1** Contributions of different components to protein production and concentration noise. Values are given as variances (squared noise) because then individual contributions can be added up. Dataset of main text (e.g. Fig. 1), strain MG22 grown on M9 + 0.1 % maltose + 200 μM Iptg.

**Supplementary Figures**


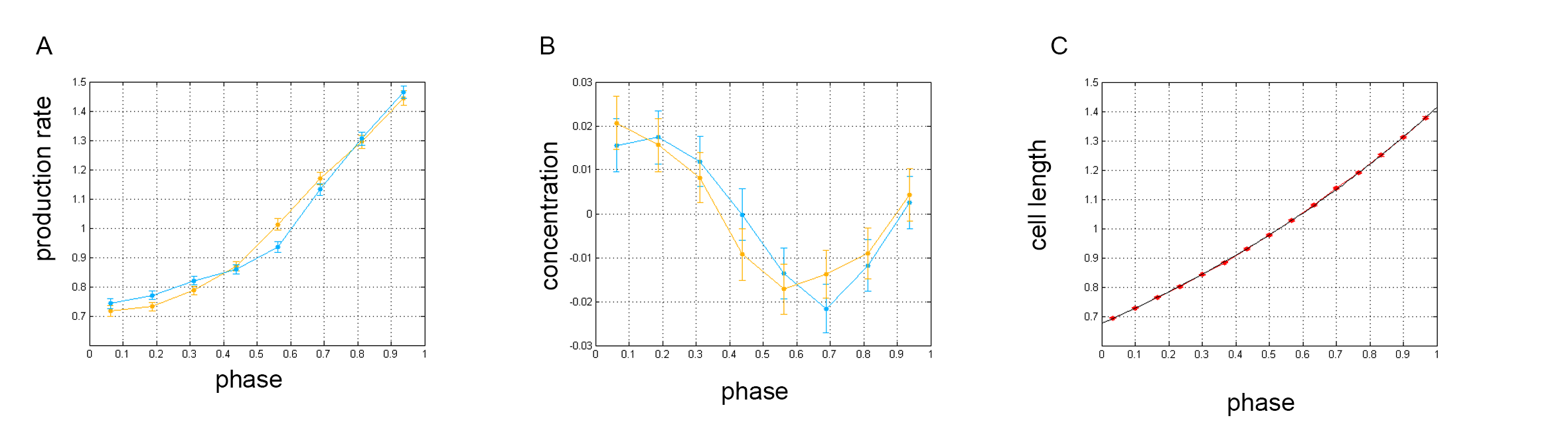


**Figure S1** Population average cell-cycle dependencies for a replicate of the dataset from the main text. Figures are to be compared to Fig. 1 and Fig. 4a,b. yellow: YFP (Fig. 1), cyan: CFP (Fig. 4)

**
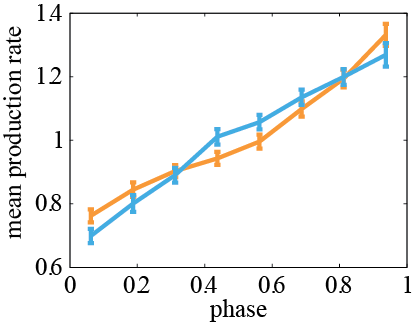
**

**FIGURE S2** Dependence of average YFP (orange line) and CFP (blue line) production rate on cell cycle phase in rich medium. Data is obtained from 215 cells with complete cell cycle, strain is MG22. Error bars are obtained by bootstrapping.


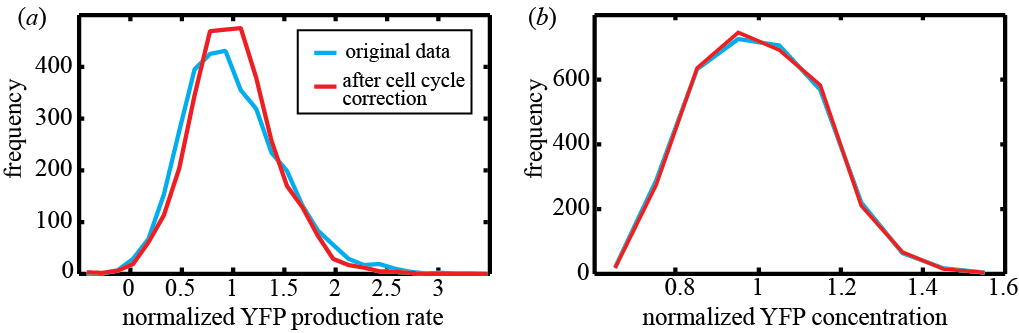


**FIGURE S3** Distribution of normalized YFP production rates (**a**) and concentrations (**b**). Histograms before subtraction of cell-cycle trends are shown in blue, histograms after subtraction are plotted in red. Variation in production rates is reduced while concentration distribution is almost unchanged.


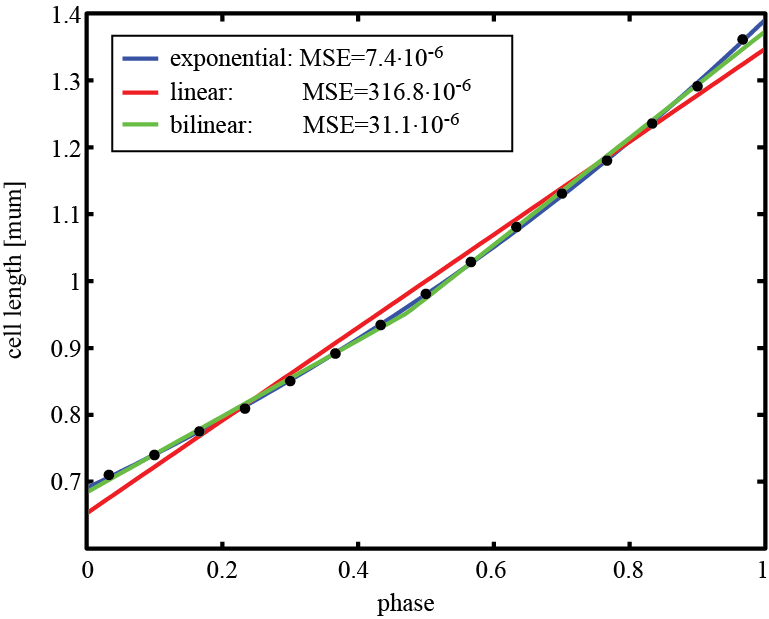


**FIGURE S4** Different growth functions are fitted to the binned phase dependence of cellular length (black dots): Tested fit functions are: exponential growth (blue line), linear growth (red line) and bilinear growth (green line). The exponential fit is best and data points deviate the most from a linear fit.


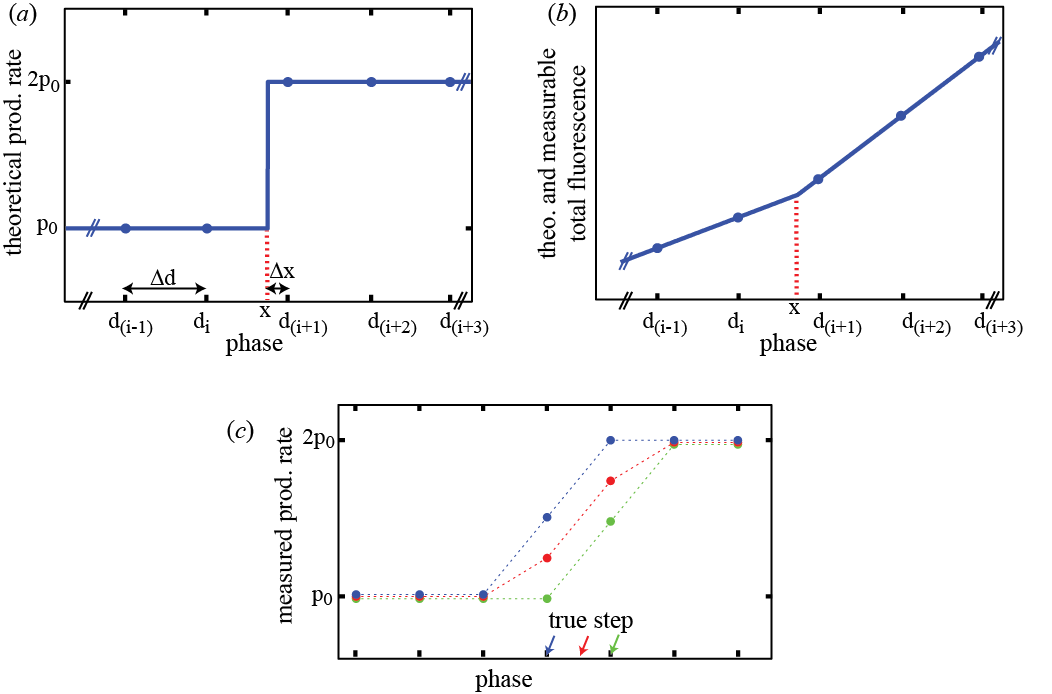
**FIGURE S5** Influence of production rate determination method on a step-like increase in production rate. (**a**) The theoretical production rate doubles instantaneously (blue line). Dots denote the time points at which production rate will be measured in an experiment, the vertical axis is however not directly measurable due to finite time delays between acquired images. (**b**) Total fluorescence (line) is the integrated production rate. For production rate calculation, total fluorescence is measured at the indicated phase points (dots). (**c**) Inferred production rate from total fluorescence data for an idealized step-like input rate. Production rate is smoothened and contains one or two data points at intermediate rates. The applied fit function in the main text corresponds to the blue and green lines, i.e. when the step occurred at one of the measured time points.

**
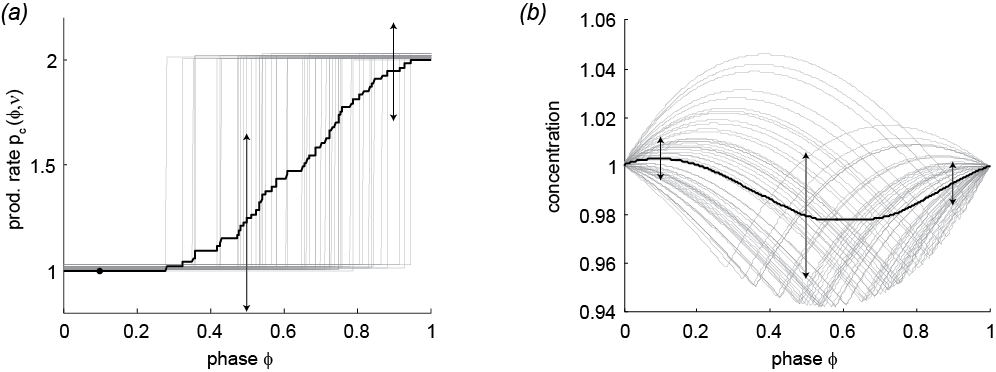
**

**Figure S6** Determination of protein noise caused by stochastic replication timing. (**a**) Production rate. Fitted step-like production rate functions, for 53 individual cell cycles (thin gray lines), and the resulting averaged production rate (black line, same as orange line in Fig. 3d). Arrows indicate three examples of the conditional variance at three phases (size corresponds to standard deviation of the single-cell traces at that phase, first arrow has length zero). (**b**) Concentration. We estimated the concentration traces (thin gray lines) using the production rate traces from (a) and dilution due to volume growth. The thick black line is the average of all single-cell traces. The stochastic and deterministic cell-cycle contributions to the concentration fluctuations were then determined in the same way as for production rate, using variance decomposition. Specifically, the conditional variance (the variance of the protein concentration at a particular phase) is displayed for three example phases (arrows). Resulting noise contributions are found in table S1. Initial values are here normalized to 1.

**
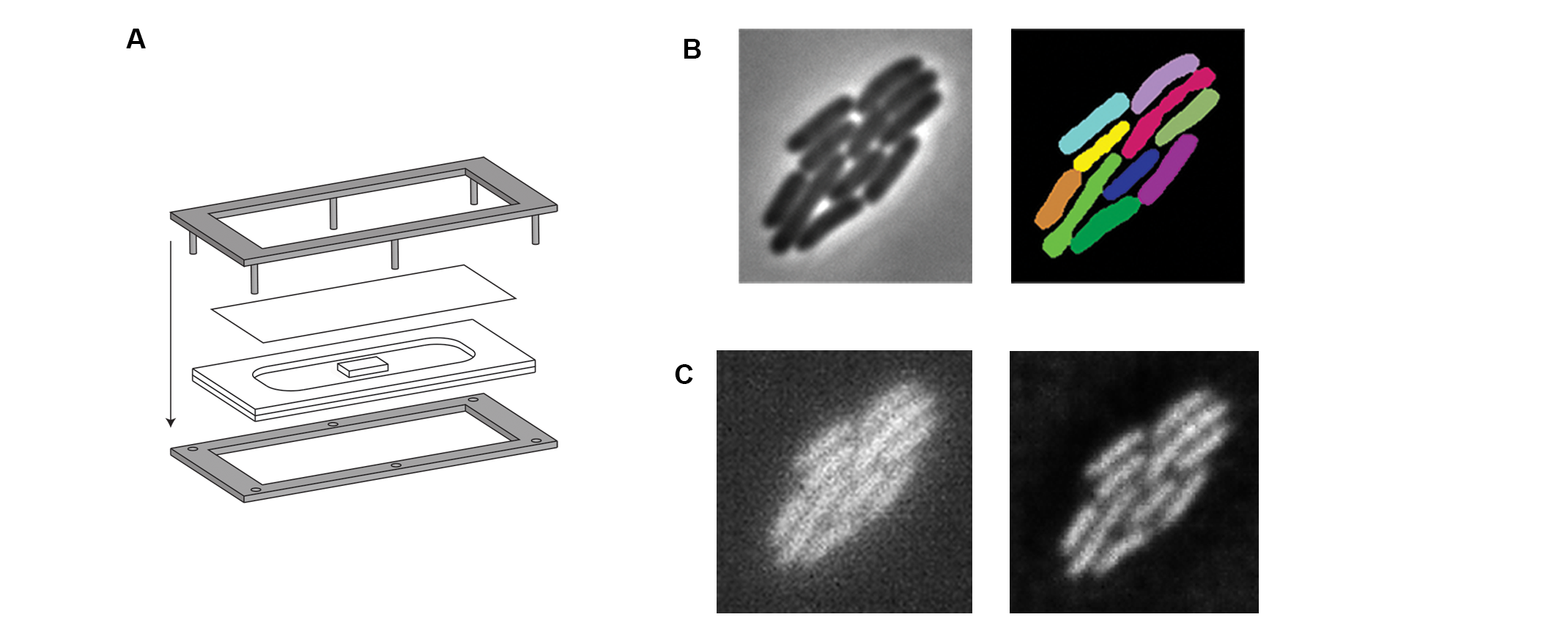
FIGURE S7** Microscopy and image analysis. (**a**) Sample preparation. An acryl gel (small rectangular shape) is placed into a glass cavity (white) and cells are pipetted onto the gel. The cavity is closed with a coverslip (white), clamped with a metal holder (gray) which is then screwed rather tight. For microscopy the sample is turned upside down. (**b**) Automated cell segmentation based on phase contrast images. (**c**) Raw fluorescent images (left) are corrected for background, uneven illumination and blurring (right). See Methods for more details.


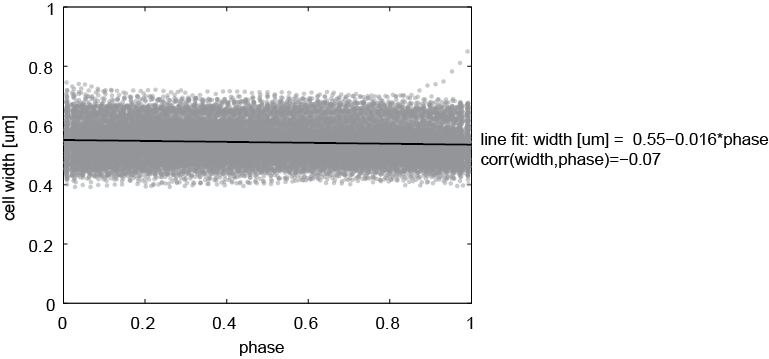


**FIGURE S8** Cell width vs. the cell-cycle phase. The width is found to be independent of cell-cycle phase.


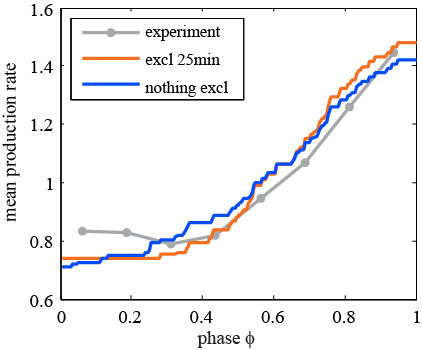


**FIGURE S9** Comparison of binned experimental population average production rate (gray) to mean of ideal step functions (orange and blue). Orange: In the fitting procedure, the step-moment in production rate is restricted to occur later than 25 minutes after cell birth. In the first 25 minutes after cell birth, fitting is less reliable. Blue: Step-moment is not restricted in time. Orange and gray curves are identical to curves in Fig. 3d of the main text.

**Supporting References**

1. Cooper S: **What Is the Bacterial Growth Law during the Division Cycle?** *J Bacteriol* 1988, **170**:5001–5005.

2. Cooper S: **Distinguishing between linear and exponential cell growth during the division cycle: single-cell studies, cell-culture studies, and the object of cell-cycle research.** *Theor Biol Med Model* 2006, **3**:10.

3. Mir M, Wang Z, Shen Z, Bednarz M, Bashir R, Golding I, Prasanth SG, Popescu G: **Optical measurement of cycle-dependent cell growth.** *Proc Natl Acad Sci U S A* 2011, **108**:13124–9.

4. Kubitschek HE: **Increase in cell mass during the division cycle of Escherichia coli B/rA.** *J Bacteriol* 1986, **168**:613–8.

5. Kubitschek HE, Pai SR: **Variation in Precursor Pool Size during the Division Cycle of Escherichia coli : Further Evidence for Linear Cell Growth**. *J Bacteriol* 1988, **170**:431–435.

6. Reshes G, Vanounou S, Fishov I, Feingold M: **Cell shape dynamics in Escherichia coli.** *Biophys J* 2008, **94**:251–64.

7. Kubitschek HE: **Bilinear cell growth of Escherichia coli.** *J Bacteriol* 1981, **148**:730–3.

8. Bipatnath M, Dennis PP, Bremer H: **Initiation and Velocity of Chromosome Replication in Escherichia coli B / r and K-12**. *J Bacteriol* 1998, **180**:265–273.
